# Detector system dose verification comparisons for arc therapy: couch vs. gantry mount

**DOI:** 10.1120/jacmp.v15i3.4495

**Published:** 2014-05-08

**Authors:** Arjunan Manikandan, Biplab Sarkar, Maitreyee Nandy, Chandra Sekaran Sureka, Michael S. Gossman, Nadendla Sujatha, Vivek Thirupathur Rajendran

**Affiliations:** ^1^ Department of Radiation Oncology NRI Medical College & General Hospital Guntur India; ^2^ Dept of Radiotherapy AMRI Hospitals Kolkata India; ^3^ Saha Institute of Nuclear Physics Kolkata India; ^4^ Department of Medical Physics School of Physical Sciences, Bharathiar University Coimbatore India; ^5^ Regulation Directive Medical Physics® Flatwoods KY USA; ^6^ Tri‐State Regional Cancer Center Ashland KY USA; ^7^ Department of Radiotherapy GGH Guntur India; ^8^ Department of Radiation Oncology Jupiter Hospital Mumbai India

**Keywords:** array, EPID, MLCPE, QA, VMAT

## Abstract

The aim of this study was to assess the performance of a gantry‐mounted detector system and a couch set detector system using a systematic multileaf collimator positional error manually introduced for volumetric‐modulated arc therapy. Four head and neck and esophagus VMAT plans were evaluated by measurement using an electronic portal imaging device and an ion chamber array. Each plan was copied and duplicated with a 1 mm systematic MLC positional error in the left leaf bank. Direct comparison of measurements for plans with and without the error permitted observational characteristics for quality assurance performance between detectors. A total of 48 different plans were evaluated for this testing. The mean percentage planar dose differences required to satisfy a 95% match between plans with and without the MLCPE were 5.2% ± 0.5% for the chamber array with gantry motion, 8.12% ± 1.04% for the chamber array with a static gantry at 0°, and 10.9% ± 1.4% for the EPID with gantry motion. It was observed that the EPID was less accurate due to overresponse of the MLCPE in the left leaf bank. The EPID always images bank‐A on the ipsilateral side of the detector, whereas for a chamber array or for a patient, that bank changes as it crosses the ‐90° or +90° position. A couch set detector system can reproduce the TPS calculated values most consistently. We recommend it as the most reliable patient specific QA system for MLC position error testing. This research is highlighted by the finding of up to 12.7% dose variation for H/N and esophagus cases for VMAT delivery, where the mere source of error was the stated clinically acceptability of 1 mm MLC position deviation of TG‐142.

PACS numbers: 87.56.‐v, 87.55.‐x, 07.57.KP, 29.40.‐n, 85.25.Pb

## I. INTRODUCTION

Volumetric‐modulated arc therapy (VMAT) is a rotational intensity‐modulated radiation therapy (IMRT), where the dose rate, gantry speed, and leaf positions of a multileaf collimator (MLC) vary during gantry rotation.^(1)^ VMAT is superior to IMRT, since VMAT is designed to provide the intended dose distribution to a total volume rather than to mere axial sections. This kind of rotational IMRT has received tremendous interest in recent years.^2^, ^3^ However, the application of such advanced treatment techniques in research has been limited by the inability of most linear accelerators to accommodate this modality. Here, we have examined one of the major practical concerns: quality assurance verification of dose delivery with regard to MLC loci. Like IMRT, VMAT requires MLC leaf position precision. Small segmented fields are required to create a homogeneous dose distribution inside the target volume. MLC leaf position tolerances for static step‐and‐shoot and dynamic delivery have already been defined in several studies.^4^, ^5^, ^6^, ^7^ Tolerances can depend on dose delivery, algorithms for modeling, and measurement methods. Each linear accelerator should be evaluated for its systematic and random inaccuracies in MLC position.^8^, ^9^, ^10^, ^11^, ^12^, ^13^, ^14^, ^15^, ^16^, ^17^, ^18^

The growing detector preference is to use an electronic portal imaging device (EPID). It has a high resolution at nominally 400 μm. However, with the EPID dosimetry package being considerable in cost, VMAT quality assurance using alternative equipment is quite common. Still, few investigators have reported systematic and random MLC positional errors (MLCPE) during arc therapy delivery or implications of it with regard to the dose distribution^18^, ^19^ Planar dose distribution analysis for this measure have been proposed, but never evaluated on an EPID.^20^, ^21^, ^22^, ^23^, ^24^, ^25^ Only a few investigators have even edited MLC log file values for leaf position as a part of test strategy.^26^, ^27^, ^28^, ^29^, ^30^

Literature review reveals disagreement for a unanimous, solitary, clinically acceptable MLC positioning error amongst researchers. MLC positional accuracy for dynamic MLC (DMLC) and static MLC (SMLC) IMRT delivery was published by various groups and reported differently with a maximum tolerability.^22^, ^29^ Experimental research was published by one group with an observed error of 2.1–3.0 mm on average and up to a maximum of 4 mm.^6^, ^7^ The American Association of Physicists in Medicine (AAPM) Task Group (TG‐142) specifies MLC calibration tolerance as 1 mm.^7^, ^14^, ^27^ Although this value was based primarily on manufacturer specifications, in order to stay in‐line with formal guidance from the AAPM we have chosen to utilize the recommended 1 mm clinical tolerance in this study as recommended.

The principal aim of this study was to evaluate the characteristics of different patient‐specific quality assurance systems during VMAT delivery, using an intentionally applied MLC positioning error within the MLC input files. We report the results of VMAT testing using a gantry stationary detector system (electronic portal imaging device or EPID) and a couch stationary detector system (ion chamber array), commonly referred to as a GS system or a CS system, with the assistance of manipulated MLC input files to purposefully introduce a 1 mm error for clinically planned cases. Results are related to tolerances of clinical acceptability, as provided by AAPM Task Group 142.^(14)^ This research will assist the medical physicist in the appropriate selection of patient‐specific quality assurance (QA) systems for a qualitative and quantitative assessment of acceptable MLC positional errors and clinical relevance.

## II. MATERIALS AND METHODS

### A. Phantom and detector system

**Figure 1:**
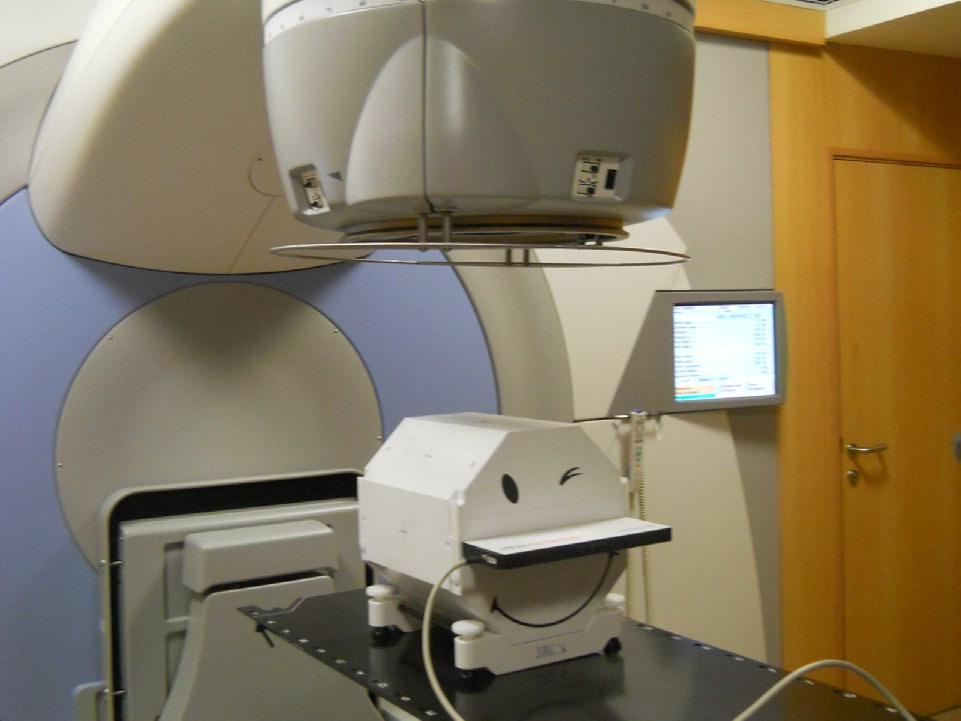
Ion chamber array and Octavius II phantom setup for VMAT dose delivery measurement.

Two different detector systems were used in this experiment. First used was an Elekta (Stockholm, Sweden) EPID (iView GT) AmSi flat‐panel imager equipped with a detector panel from Perkin‐Elmer (Fremont, CA). The pixel resolution specification is 400μm for a matrix dimension of 1024×1024. The source‐to‐detector distance was 160 cm with active imager dimension of $25.6\times 25.6\;{\text{cm}}^{2}$ at isocenter for all measurements. Above the imager is a copper plate with $133\;\text{mg}/{\text{cm}}^{2}$ terbium‐doped gadolinium oxysulfide added as a buildup material. Second, an ion chamber array from PTW (Freiburg, Germany), Model Seven29, was used. Seven29 is a large area pixel segmented ion chamber array containing 729 vented cuboid ionization chambers. The detector size is 0.5cm×0.5cm×0.5cm (0.125 cc) with a center‐to‐center detector separation of 1 cm. The maximum measureable field size is $27\times 27\;{\text{cm}}^{2}$. The Seven29 was used along with a PTW OCTAVIUS II phantom. This phantom adapts the 729 ion chamber array within. Data were compiled and analyzed using PTW Verisoft software. The ion chamber and phantom setup is shown in Fig. 1.

### B. Dosimetric study

VMAT plans were generated in an Elekta Monaco treatment planning system (TPS) version

**Figure 2:**
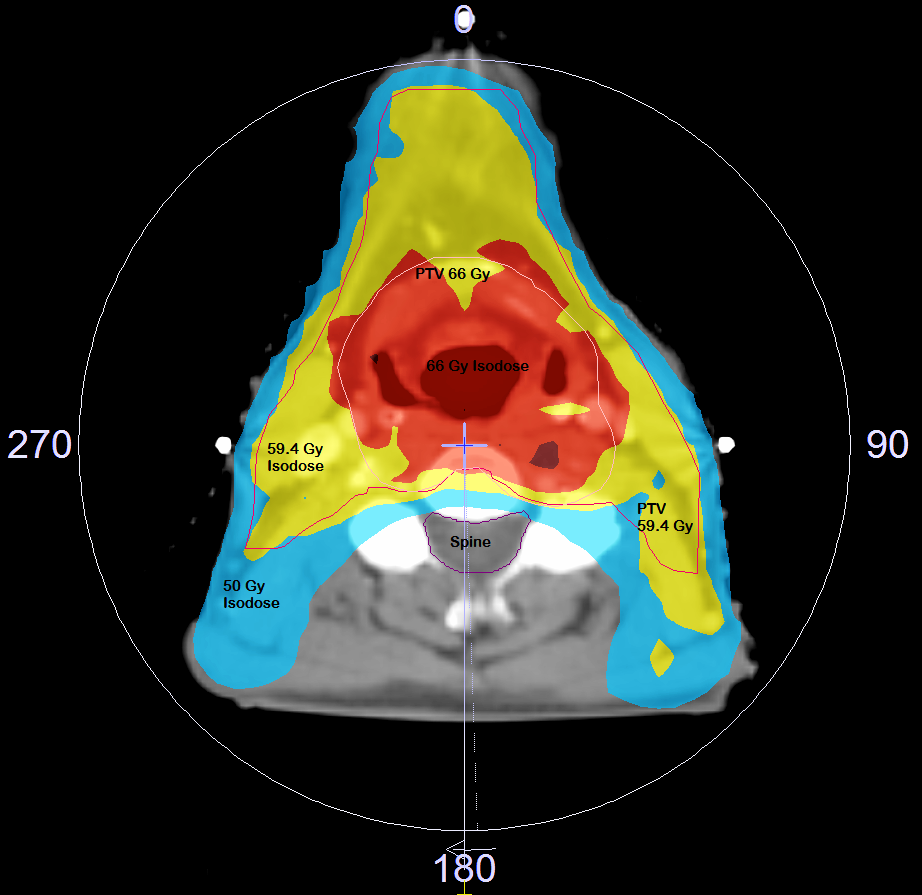
Isodose distribution for head and neck case using a single full arc VMAT plan. Treatment plan generated for 66 Gy in 33 fractions and 59.4 Gy in 33 fractions

2.3.1, commissioned to operate using the X‐ray Voxel Monte Carlo Algorithm for the Elekta Synergy S linear accelerator. In order to challenge the detector systems at the highest level, we chose clinical plans with high‐dose gradients. As such, plans for patients with head and neck (H&N) cancer or esophageal cancer were ideal. H&N planning involves structures of high density, such as the mandible, teeth, and skull base, while esophageal targets contain low‐density volumes like the trachea, carina, and the lung. Four H/N and four esophagus patient plans were chosen for the study. Each clinical treatment plan incorporated a single arc for therapy, designed to achieve clinical doses of 180–200 cGy per fraction. An axial section of head and neck single arc VMAT plan is shown in Fig. 2. For investigational purposes, each plan was saved as a text file and copied. The duplicate plan was then systematically introduced with a 1 mm multileaf collimator positional error in bank‐A (left side) for all leaves. Direct comparison of measurements for plans with and without the MLC positional error would permit quality assurance performance characteristics to be observable between detector types. As planned, radiation delivery was produced through the use of an Elekta Synergy S linear accelerator equipped with an MLCi2 model collimation system comprising of 40 pairs of 1.0 cm projected MLC leaves.

By editing the MLC input file positions for bank‐A leaves using an in‐house Visual Basic program, the newly modified plan was identical, yet with bank‐A leaves 1 mm further open. Both the original plan without MLCPE (set I termed “MLC0PE”) and the modified plan with a systematic MLCPE (set II termed “MLC1PE”) were each copied, permitting generation of new plans again with disabled arc rotation. In this manner, all IEC 61217 gantry angles were kept static at 0° for downward delivery.^(7)^ The consequence of dose variation existing between these two sets of plans was the focus of study.

Starting with eight original plans and duplicating each with a 1 mm MLC error creates eight more plans for a total of 16. These were then copied and duplicated again with disabled gantry motion, resulting in 32 plans. However, with the couch stationary detector system, measurements were obtained for dynamic gantry delivery as well as for downward static gantry delivery. Therefore, 16 more copied plans were introduced for a net of 48 plans for evaluation by measurement. The schedule of delivery thus included measurements with the gantry rotating for 16 plans, each using the CS (couch stationary) system and the GS (gantry stationary) system. This was followed by measurements with the gantry angle fixed to $G\theta =0^{\circ }$ for all beams in 16 additional plans using only the CS system The GS detector is stationary in space relative to the source as it is affixed to the gantry.

**Figure 3:**
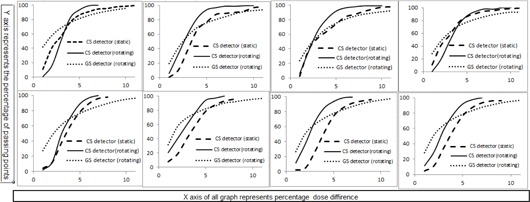
Plan testing results for eight VMAT plans: four esophagus plans (P1‐P4) and four H/N plans (P5‐P8) with and without a 1 mm MLC positioning error; measurements were obtained by the CS detector for arc therapy, as well as collapsed static gantry delivery; measurements were obtained by the GS detector for arc therapy only.

During VMAT delivery, the GS detector maintains a constant geometrical relationship with the radiation source and the MLC, since it rotates with the gantry. The couch stationary detector system was the PTW OCTAVIUS II phantom, containing a 729 ion chamber array, monitored with PTW Verisoft software. The chamber array similarly maintains a constant geometric relationship with the radiation source and MLC, although mounted on the couch. After irradiation, coronal plane doses were evaluated for differences between the MLC0PE and MLC1PE plans for both detector configurations. Figure 3 illustrates the variation of matched points between MLC0PE and MLC1PE deliveries with varying percentage dose difference only. Eight panels indicate eight patients, each with three curves indicating the percentage of matched points measured by the CS system enabling and disabling gantry rotation and the GS detector with enabled gantry rotation.

The percentage relative dose differences were taken as the difference between coronal plane passing results for the delivery without MLC error to that having the 1 mm error introduced $\left(D_{\text{MLC}0\text{PE}}-D_{\text{MLC}1\text{PE}}\right)$. The Van Dyke criteria of percentage dose variation agreement was incorporated to compare the dose distribution differences between MLC0PE and MLC1PE plans.^(31)^ Specifically, the percentage dose difference was evaluated instead of an evaluation of a gamma index which consists of both percentage dose difference and distance to agreement (DTA). It was known *a priori* that leaf position errors should be measured by maintaining identical detector positions (DTA=0).^(19)^ Our accepted tolerance of 95% points passing was tabulated for analysis.^(31)^ The results for all plans from all detector systems are presented in Table 1. For completeness, we note the gamma analysis result for the couch stationary detector having rotational gantry measurement for the plans with and without MLCPE in Table 2. Table 2 yields the mixed results of high and low gamma passing ranges at 2%‐2 mm and 3%‐3 mm DTA and ADD.

**Table 1:** Cumulative plan testing results and statistics for eight VMAT plans: four esophagus plans (P1‐P4) and four H/N plans (P5‐P8) with and without a 1 mm MLC positioning error. Relative dose difference between MLC0PE vs. MLC1PE to achieve 95% matching rate. $G\theta =0^{\circ }$ indicates measurements with a static gantry delivery; ΔGθ indicates arc therapy delivery

	*QA plan: CS detector* $\left(G\theta =0^{\circ }\right)$			*QA plan: CS detector* (ΔGθ)			*QA plan: GS detector* (ΔGθ)			*Patient Plan: Actual* (ΔGθ)		
	*Esophagus*		*H/N*	*Esophagus*		*H/N*	*Esophagus*		*H/N*	*Esophagus*		*H/N*
Patient 1	‐		6.8	‐		5.1	–		10.0	‐		6.5
Patient 2	‐		7.8	‐		5.4	‐		10.5	‐		6.9
Patient 3	‐		8.4	‐		5.7	‐		9.8	‐		7.4
Patient 4	‐		7.6	‐		5.0	‐		9.8	‐		6.4
Patient 5	7.5		‐	5.6		‐	9.5		‐	5.1		‐
Patient 6	9.6		‐	5.4		‐	12.5		‐	5.2		‐
Patient 7	9.7		‐	4.8		‐	12.7		‐	5.2		‐
Patient 8	7.5		‐	4.3		‐	12.4		‐	4.6		‐
Mean(4P)	8.6		7.7	5.0		5.3	11.8		10.0	5.1		6.8
SD(4P)	1.2		0.7	0.6		0.3	1.5		0.3	0.3		0.5
Mean(8P)		8.1			5.2			10.9			5.9	
SD (8P)		1.0			0.5			1.38			1.0	

P=patient.

**Table 2:** Measured dose distributions are compared with and without leaf position error, using gamma analysis for couch stationary detector in rotational gantry measurements

	*Passing Points for 2mm/2% Gamma*	*Passing Points for 3mm/3% Gamma*
Esophagus Patients	61.6	78.2
	87.0	97.7
	73.3	96.6
	72.5	94.4
Head and Neck	53.1	81.8
	64.4	92.8
	58.8	89.2
	53.0	81.5

### C. Verification measurement

The treatment planning system was held as the basis for evaluation. The planning software was modeled to include precise positioning of the MLC. The coronal planes from the actual patient plans with and without MLC positional error was transferred to the Verisoft software and used for comparison in similar to measured data. The relative percentage dose difference was varied to achieve the correlation graph between it and matching points between MLC0PE and MLC1PE. A strict comparison of results from arc delivery to those obtained with a static gantry is the best method for identification of any systematic error. It is not possible to use Elekta Monaco software in commercial form to program a collapse of all the gantry angles to 0° for a VMAT plan. In order to achieve this, changes were made in the source code of Monaco treatment planning system. With each beam then having been forced to a static downward delivery, there was absolutely no chance for any error to be introduced by rotational motion within the plan. It then followed that the comparison between two planar dose distributions from the chamber array represents the true characteristic behavior of the MLC during irradiation. The values are tabulated in the Table 1.

### D. clinical study

In order to evaluate the clinical significance of the 1 mm MLC positional error acceptability specified in TG‐142, we compared measured results from clinical patients with and without a 1 mm MLCPE using dose‐volume histogram analysis, as detailed in Table 3.^(14)^ The experiments were specific for H/N cases with contoured structures including the planning target volume (PTV), right and left parotid arteries, spinal cord, and the larynx. Doses were evaluated for both MLC0PE and MLC1PE plans. Parallel structures, like the parotid and larynx, were evaluated for mean organ dose. Other structures were evaluated for the maximum dose imparted to 1% of the volume. Esophageal cases were evaluated for mean dose to both lungs and heart, as well as for the volume receiving 20 Gy and 50 Gy, respectively. In all cases, the PTV was evaluated for the tumor volume dose coverage (dose received by 95% tumor volume) and a hot spot reference (volume receiving at least 107% of the prescription dose).

**Table 3:** DVH analysis for eight patients detailing the dose difference between plans with and without MLCPE

*Head and Neck*													
		*Patient 1*			*Patient 2*			*Patient 3*			*Patient 4*		
*Tumor/OAR*	*Dosimetric Parameter*	*MLC0PE (cc)*	*MLC1PE (cc)*	*Relative (%) Difference*	*MLC0PE (cc)*	*MLC1PE (cc)*	*Relative (%) Difference*	*MLC0PE (cc)*	*MLC1PE (cc)*	*Relative (%) Difference*	*MLC0PE (cc)*	*MLC1PE (cc)*	*Relative (%) Difference*
PTV	Dose Coverage (D95%)	246.0	251.9	‐2.4	179.2	182.6	1.9	203.8	208.4	5.7	183.9	189.8	3.2
	Hot Ref (D107%)	10.3	42.2	12.4	4.1	20.7	8.9	5.5	30.1	11.4	0.8	3.8	10.5
RT PAROTID	MEAM (Gy)	38.3	39.8	3.9	22.2	23.1	4.1	32.2	33.7	4.7	27.3	28.4	4.0
LT PAROTID	MEAN (Gy)	36.6	38.2	4.4	24.3	25.4	4.5	28.8	30.3	4.2	28.7	30.3	5.6
SPINE	1% volume	42.3	43.9	3.8	40.4	42.1	4.2	40.2	41.7	3.7	41.4	43.2	4.4
LARYNX	MEAN (Gy)	42.9	44.6	4.0	43.8	45.3	3.4	53.6	58.5	9.1	42.1	43.6	3.6
*Esophagus*													
		*Patient 5*			*Patient 6*			*Patient 7*			*Patient 8*		
*Tumor/OAR*	*Dosimetric Parameter*	*MLC0PE (cc)*	*MLC1PE (cc)*	*Relative (%) Difference*	*MLC0PE (cc)*	*MLC1PE (cc)*	*Relative (%) Difference*	*MLC0PE (cc)*	*MLC1PE (cc)*	*Relative (%) Difference*	*MLC0PE (cc)*	*MLC1PE (cc)*	*Relative (%) Difference*
PTV	Dose Coverage (D95%)	351.9	333.0	5.0	386.4	369.5	4.4	246.5	241.4	2.0	206.5	203.1	1.6
	Hot Ref (D107%)	4.2	47.3	11.4	8.2	39.1	7.4	4.5	28.2	9.5	8.1	15.8	3.8
RT LUNG	MEAN	27.5	26.8	2.7	15.6	15.1	3.5	12.1	11.8	2.5	20.8	20.1	3.8
	V20Gy	66.3	64.3	3.1	25.6	24.1	6.1	8.9	8.0	10.7	37.7	35.1	7.5
LT LUNG	MEAN	13.9	13.4	3.8	15.7	15.1	3.5	15.4	14.9	3.3	17.3	16.6	4.5
	V20Gy	14.8	13.2	11.6	32.3	30.6	5.4	27.3	26.0	5.2	30.3	27.7	9.7
HEART	MEAN	24.0	23.4	2.6	24.2	23.3	3.6	27.4	26.7	2.5	27.7	26.7	3.6
	V50Gy	9.2	9.5	2.6	12.4	11.2	10.7	9.2	8.6	7.5	6.8	8.0	14.5

## II. RESULTS

### A. Dosimetric study

The mean dose difference to achieve 95% matching between the plans with and without MLC error obtained by the chamber array for rotational measurements for all eight cases were 5.2%±0.5%, as shown in Table 1. For individual H/N and esophagus cases, the results were 5.3%±0.3% and 5.0%±0.6% respectively. For the GS detector, the mean dose differences were 10.9%±1.4% for all plans, 10.0%±0.3% for H/N plans, and 11.8%±1.5% for esophagus plans. Less appreciable results were observed for static gantry delivery to the array. Mean dose difference for esophagus, H/N, and combined all eight patients were 8.6%±1.2%,7.7%±0.7%, and 8.1%±1.0%, respectively.

**Figure 4:**
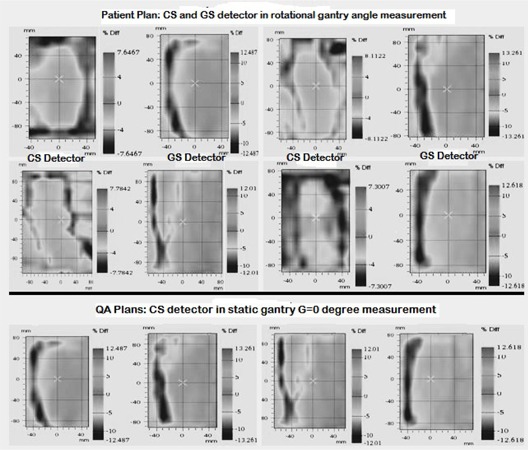
Energy fluence map results detailing the dose distribution percentage relative difference (MLC0PE‐MLC1PE) for two H/N plans and two esophagus plans with and without the 1 mm MLC error; (top) array and EPID results for arc rotation delivery; (bottom) array results for static gantry delivery.

The variation in dose distribution is qualitatively observed in Fig. 4. There, the top two rows showed the GS detector and CS detector in rotational gantry measurements side‐by‐side for four patients and the bottom row featuring the CS detector static gantry measurements for the same four patients. The result obtained in GS detector arc measurements is more than twice that obtained by the CS detector. For the GS detector, failing points in the dose distribution are markedly present in the left side for all studied patients. This is directly attributed to the fact that the left bank was introduced with an MLC positional error. The GS detector was able to discern the error consistently, whereas the CS detector showed a more uniform result.

The ability of the GS detector to identify the error more readily is due to the higher resolution of the panel. With a resolution of 400μm, the EPID is able to identify dose gradients in fine detail, which then correlates to more accurate results. Using Table 1, we were able to distinguish the rotational error magnitude as measured by the CS detector. Subtraction of the mean variation for all eight plans for the CS detector during arc therapy to that with static gantry delivery results in 3.0% systematic error. No error was present for GS detector measurements, since arc therapy and static delivery yielded the same result. This research is highlighted by the finding of up to 12.7% dose variation for H/N and esophagus cases for VMAT delivery, where the mere source of error was the stated clinically acceptability of 1 mm MLC position deviation.

The gamma analysis for the CS detector in the rotational gantry measurement in Table 2 indicate a mixed result of low and high gamma passing rate for 2 mm‐2% and 3 mm‐3%. For esophagus cases, 2%‐2 mm and 3%‐3 mm gamma gives a minimum and maximum value of 61.6%, 87.0% and 78.2%, 96.6%, respectively. Similarly for head and neck cases, a 2%‐2 mm and 3%‐3 mm gamma results in a minimum and maximum value of 53.0%, 64.4% and 81.5%, 92.8% respectively.

### B. Verification measurement

The isocentric coronal plane comparisons for 95% points matching patient plans with and without MLCPE in rotational gantry are showed in Table 1. Quantitative and qualitative analysis of the results revealed more similarity between planned results and measured results for the CS system in rotational gantry. Treatment plans were calculated with a grid resolution of 3 mm. Verisoft measurement software utilizes the same resolution. However, the detectors are spaced 1 cm apart, relieving the accuracy of the results for the CS system in rotational measurement. Although there is a significant difference in the grid resolution between the TPS and the CS system, results show that such arrays have an ability to discern even the stated clinically acceptable MLC positional error. TPS measurements are free of any mechanical uncertainty like random MLC positional error, gantry angle, and systematic shifts in detector placement using LASER. A CS detector in rotational gantry measurements produces almost the same result as that of TPS measurement.

### C. clinical study

The dose‐volume histogram analysis in Table 3 reveals the dose difference between the plans with and without MLCPE. The mean variation of tumor dose coverage and the hot spot reference for the esophagus structure was 3.4%±1.7% and 8.0%±3.3%, respectively. For H&N cases, these were 3.3%±1.7% and 8.5%±4.9%, respectively. The minimum and maximum dose differences were obtained for the same patient (esophagus Patient #4). The minimum percentage difference was noted as 1.6% for tumor dose coverage. The highest difference was noted as 14.4% for a heart volume receiving 50 Gy dose. It was observed that, for all cases, hot spot increases significantly on introduction of the MLC positional error to the plan.

## III. DISCUSSION

Different investigators have characterized the variation of the dose distribution induced by poor MLC position in terms of errors that are either systematic, random, or both.^12^, ^29^, ^30^ One group conducted a study using Varian Medical Systems, Inc. (Palo Alto, CA) Eclipse software for RapidArc delivery, showing target dose uncertainty remains within 2% for 0.6 mm systematic MLCPE.^(18)^ For Elekta VMAT using the MONACO treatment planning system, one group reports 2% and 3% dose difference yields the average pass rate when compared between with and without MLCPE were 68.9%±11.4% and 91.5%±5.1%, respectively, obtainable with ±1mm systematic MLCPE.^(19)^ Our report extended this finding to above 10% for GS detector, and nearly 8% and 5% for CS detector in static and rotational gantry measurements. No other significant study is found relating to this type of testing, while incorporating MLC input file positioning error modifications.

We associate findings from other research on IMRT delivery to illustrate the interest of MLC error causations. As an example in one study, a 1 mm systematic MLCPE change resulted in target coverage changes by 8% in complex plans.^(12)^ Another group reported a random error of 2 mm gave a negligible dose discrepancy, whilst a systematic MLCPE of 0.3 mm gave a dose uncertainty less than 2%.^(29)^ A leaf‐to‐leaf gap error was discussed by only one group which showed that, for a 1 mm gap, the energy fluence can be altered by up to 6%.^(13)^ According to TG‐142, a 2 mm MLCPE is clinically unacceptable.^(14)^ Still, researchers continue to state clinically insignificant doses are observed when detector arrays are used.^12^, ^29^ The above discussion accentuates the sensitivity of IMRT/VMAT dose delivery being susceptible to systematic errors, but not from a random MLCPE. The reason for this occurrence is now apparent from our investigations.

**Figure 5:**
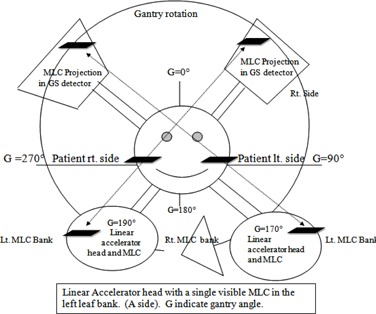
Schematic for geometrical relationship between the gantry, MLC, patient or CS detector, and GS detector for VMAT delivery; one MLC of left leaf bank is depicted here. At gantry angle 190° and 170° MLC from left bank projecting on the right side and left side, respectively, of the CS detector/patient. Same MLC projecting at the left side of the GS throughout the rotation. MLC direction changes for patient and CS detector when G0 crosses ($-90^{\circ }$ and 90°); for GS detector, this direction change is not applicable.

We have introduced a 1 mm MLC bank‐A leaf positional error and taken measurements with a GS (gantry stationary) detector system and a CS (couch stationary) detector system. As the portal imager GS detector is rotating at the same speed with the gantry (radiation source & collimation system), an MLC positional error on the left side will always appear to be on the left side of the imager when the gantry rotates clockwise from $-180^{\circ }$ through to $+180^{\circ }$. The lateral direction of the effect will always be the same. Thus, the dose difference reveals this errant accumulation on the left side. In the case of a couch stationary detector, the effect resulting from the MLC positional error will reverse its direction as the gantry crosses $-90^{\circ }$ and the $+90^{\circ }$ position during rotation. This gives rise to a dose error, since the MLC positioning error is uniformly distributed over the irradiated area. Figure 5 explains why this happens schematically. Using standard linear accelerator directional nomenclature, the right side of a head towards gantry supine patient is denoted the A direction and the left side is called B direction. During VMAT delivery, the gantry is moving from $-170^{\circ }$ in a clockwise direction to $+170^{\circ }$. With MA representing the MLC in A side, while the gantry is at $-170^{\circ }$ we see that MA is on the right side of the patient (MR) and A side of the GS detector EPID. If without changing the MLC position, the gantry is rotated to $+170^{\circ }$, the MLC is still on the A side of the MLC head and GS detector. Yet, for the patient, it is on the left side (ML). It is evident that the directional change of the MLC does not occur for a gantry stationary detector system, such as the EPID. However, the change does occur for couch stationary detectors, such as the chamber array, just as it does for the patient. This justifies the one‐sided error accumulation of for GS detector systems. The error in dose distribution, counted by GS detector system, is attributed to its geometrical position against MLC bank and therefore virtual. This over counted error will not contribute to the actual treatment delivery in case of similar situation during patient treatment.

This finding of this experiment is governed by the geometrical relationship between the detector placement and radiation head; therefore, it is not affected by the number of treatment arcs, collimator angle, and table angle. A GS detector is independent of table angle as it is moving in synchronous motion with gantry head. However, if collimator angle is taken as a variable during the arc therapy delivery it may affect the measured dose only for a GS detector, as not showing the biased accumulation of dose delivery error. However, option for considering the collimator angle as a variable during arc therapy delivery is not available with MONACO TPS.

The most reliable detector system is CS detector system, which is proven by these verification measurements. This is conclusive from two facts. First, the ion chamber array has the ability, irrespective of its resolution, to distinguish the dose difference caused by 1 mm MLCPE, as with TPS‐generated values. And secondly, fidelity of CS detector system measurements to the treatment delivery simulated by the TPS. Therefore, for proper quality assurance of dose delivery, the best possible reproduction of the patient's geometrical configuration relative to the gantry should always be incorporated.

The clinical study indicates the significance of the AAPM TG‐142 recommended MLCPE of ±1 mm.^(14)^ The dose differences found here vary from 2.6% to as high as 14.4%, with mean dose error of more than 5%. The ICRU‐recommended value of maximum allowable dose difference in all steps is 5%.^(32)^ However, the contributions of only the allowable MLCPE supersedes this set tolerance limit. Therefore, the TG‐142‐recommended MLC tolerance yields clinically significant, or rather unacceptable, dose differences.^(14)^ It may be questioned why only single MLCPE was tested. As indicated in Fig. 4, the advantage of planar dose verification by the GS detector EPID for arc therapy is that it can detect (not quantify) systematic MLC errors that are not accurately identifiable with a CS system, like a diode or chamber array, in the rotational measurement. However, it was observed that a 1 mm systematic error in one leaf bank produces a significantly higher and clinically unacceptable (>10%) dose difference, according to ICRU‐24, when measured by a gantry stationary detector system.^(33)^ The synchronized motion of the GS detector system and radiation delivery to the patient leads to a constant geometrical relationship between them. It is this relationship that influences the dose distribution measurement by this kind of detector system to be higher on one side.

As seen here, a 1 mm MLCPE proved to cause doses considerable (10.9%±1.4%) for complex plans of eight different H/N and esophagus patients, as identified with a GS detector. A CS detector reports the error as merely half (5.2%±0.5%). A CS detector in collapsed gantry angle (zero degree) generates quantitatively exactly similar results to that of a GS detector. Assessing the experimental results in geometrical perspective of MLC position, radiation head, detector placement, and motional (rotational) aspect of dose delivery and measurement it was observed that in case of actual treatment delivery, where patient is placed in the couch, can be reproduced by a CS detector system with gantry rotation as per the treatment plan (i.e., rotational gantry). This is further established by the results obtained in verification measurements using the TPS patient isocentric coronal planes. Results were quantifiably less accurate for GS detector system (or a surrogate like CS detector system in collapsed gantry angle) and suffer from systematic error, as proven with both gantry stationary and arc‐enabled VMAT delivery. Therefore, the result obtained by GS detector or CS detector in collapsed gantry is overestimating the dose delivery error, which is not applicable during actual treatment delivery to the patient.

The gamma analysis result presented in Table 2 do not reveal any quantitative orderly indication or trend in gamma failure (or pass) pattern seamlessly indicating the systematic MLCPE in the left MLC leaf bank. Furthermore, qualitative observational characteristic of the difference between MLC no error and MLC error planner gamma distribution $\left({\text{gamma}}^{\text{MLC}0\text{PE}}-{\text{gamma}}^{\text{MLC}1\text{PE}}\right)$ do not depict the bias accumulation in any specific side. Hence, difference in planner gamma $\left({\text{gamma}}^{\text{MLC}0\text{PE}}-{\text{gamma}}^{\text{MLC}1\text{PE}}\right)$ did not establish exclusively the effect of induced systematic MLC positional error and its biasness to left MLC bank. Lower gamma scoring throughout the field of irradiation can be attributed to several different mechanical and dosimetric reasons.

## IV. CONCLUSIONS

This study seamlessly accentuates the geometrical properties between the detector system and the radiotherapy unit during VMAT delivery and influence of the detector placement in delivered dose measurement. The observed result varied between the detector systems and its placement. For an arc‐enabled measurement of VMAT, a GS detector system measured twice the percentage dose delivery error than the CS detector system. A CS detector in collapsed gantry angle zero geometry generates quantitatively exactly similar result to that of a GS detector. Assessing the experimental results in geometrical perspective of MLC position, radiation head, detector placement, and motional (rotational) aspect of dose delivery and measurement, it was observed that in case of actual treatment delivery where patient is placed in the couch, results can be reproduced only by a CS detector system with gantry rotation, as per the treatment plan.

Gantry stationary detector and couch stationary detector measurements produce analogous result in the energy fluence measurement if there is no systematic MLC positional error. On the basis of observational characteristic of its measured dose distribution (MLC0PE‐MLC1PE) presented in Fig. 4, it can be concluded that a couch stationary detector system cannot qualitatively identify the intentional systematic MLC positional error in a rotational gantry measurement. This is because the dose error (MLC0PE‐MLC1PE) is patched all over the irradiated area (Fig. 4, cell number (1,1), (1,3), (2,1), (2,3)). Patching of dose error throughout the irradiated area could be caused by several other reasons, such as detector positional shift or error in dose calibration. Therefore, it cannot be categorically stated that dose error is attributed to MLCPE only. The same phenomenon can be readily identified by the CS detector in collapsed gantry angle $\left(G=0^{\circ }\right)$ measurement. The preferred methodology of the VMAT QA always in rotational gantry measurement. We also conclude from clinical assessment that the suggested maximum tolerable deviation for MLC position recommended in TG‐142 be used with caution, since the 1 mm stated clinical limit can cause a mean dose difference of more than 5% (histogram analysis). It is recommended that the newly formed TG‐198 reconsider this recommendation made in the now three‐year‐old TG‐142 publication.^(14)^
